# Twenty-seven-gauge vitrectomy: a consecutive, single-centre case series with exclusive use over a 4-year period

**DOI:** 10.1186/s12886-023-03265-w

**Published:** 2023-12-21

**Authors:** Eugene Ng, Mouayad Masalkhi, David H. Steel, Jasna Pavičić-Astaloš, Claire Nolan, Sarah Mernagh, Emmanuel Ankamah

**Affiliations:** 1 Institute of Eye Surgery, UPMC Whitfield Hospital, Butlerstown North, Cork Road, X91 DH9W Waterford, Ireland; 2 Institute of Eye Surgery, UPMC Kildare Hospital, Clane, Ireland; 3 UCD School of Medicine, University College Dublin, Belfield, Dublin Ireland; 4 Bioscience Institute, Newcastle University, Newcastle Upon Tyne, UK

**Keywords:** 27-gauge, Pars plana vitrectomy, Retinal detachment, Vitreoretinal Disease

## Abstract

**Background:**

To assess the safety and effectiveness of the exclusive use of 27-gauge instruments for all vitreoretinal diseases requiring vitrectomy.

**Methods:**

In this retrospective study, 1020 consecutive surgeries were performed on 958 eyes of 848 patients using 27-gauge instruments from March 2017 to June 2021. Patients with a minimum follow-up of 3 months were included. Surgical case-mix, best-corrected visual acuity (BCVA), intraocular pressure (IOP), intra- and post-operative complications, and surgery times were recorded.

**Results:**

The study patients were followed up for averagely 11 months. Of the 1020 vitrectomies, 958 were primary procedures. Of the 148 retinal detachment (RD) cases, 138 (93%) required a single vitrectomy. Primary macular hole closure was achieved in 143 of 145 (99%) cases. The average surgical times were 55 and 38 min for RD surgeries and for all other indications, respectively. BCVA improved significantly at the final visit (20/49) compared with the pre-operative visit (20/78) (p < 0.01). IOP was similar at the pre-operative (14.8mmHg) and final (14.3mmHg) visits. Complications recorded include transient hypotony in 39 eyes, iatrogenic retinal breaks in 2 eyes, and a vitreous bleed in 1 other eye.

**Conclusion:**

This study revealed that 27-gauge vitrectomy instruments can be used for a wide range of indications, with exclusive use in certain settings. The outcomes were similar to other gauges, including for rhegmatogenous retinal detachment, with minimal complications.

## Key messages

**What is known:**

i. 27-gauge vitrectomy is a safe and effective surgery for managing vitreoretinal diseases.

**What this paper adds:**

i. Our study showed that it is possible to use 27-gauge vitrectomy instruments for a wide range of indications, with exclusive use in certain settings as presented in this series. ii. 27-gauge vitrectomy outcomes were similar to other gauges, including for rhegmatogenous retinal detachment, with minimal complications.

## Background

The past 30 years has witnessed the development, and transition to the use, of vitrectomy instruments that result in smaller, less traumatic sclerotomies and incisions. These smaller gauge instruments ensure improved intraocular pressure control, better wound healing, minimal pain, and low rates of intraoperative and postoperative complications that include conjunctival scarring, inflammation, early postoperative hypotony, and endophthalmitis [1–5].

In 2010, Oshima and colleagues provided the safety profile of 27-gauge vitrectomy. [6] Following on from this initial study, many authors have provided further evidence to support 27-gauge vitrectomy as a safe and effective approach for all vitreoretinal indications. Of note, 27-gauge vitrectomy has been reported to reduce wound sealing-related complications and offers greater membrane dissection precision [2, 3, 7].

Despite the surge in literature on the safety and efficacy of 27-gauge vitrectomy, it appears that this instrumentation has not been widely adopted by retinal surgeons. In fact, the 2021 Preferences and Trends (PAT) Survey of the American Society of Retinal Surgeons (ASRS) has shown that 67% of surgeons who responded to the survey did not utilize 27-gauge instrumentation for any of their cases in the preceding year and only 2% of surgeons used 27-gauge instruments for 61–100% of their cases [8]. That aside, no study to date, to the best of our knowledge, has reported the exclusive use of 27-gauge vitrectomy for all indications. This retrospective, consecutive case study provides data over an extended period, in a centre where no other vitrectomy gauges were used, to assess the safety and effectiveness of 27-gauge vitrectomy.

## Methods

### Study design

Consecutive patients who underwent 3-port pars plana vitrectomy (PPV) for all vitreoretinal indications by a single surgeon from March 2017 to June 2021 at the Institute of Eye Surgery, Republic of Ireland were retrospectively entered into this non-randomized study. Anonymized data were collated from Medisoft Electronic Medical Records (Medisoft Limited, UK). Patients with follow-up less than 3 months as well as patients undergoing other vitreoretinal procedures without vitrectomy were excluded from the study.

Patient demographics and indication for vitrectomy were analysed. Intra- and post-operative complications were counted. Surgical time was averaged using medical insurance coding data and data was divided into only 2 groups of surgical indication (either retinal detachment surgery or non-retinal detachment vitrectomies).

### Pars plana vitrectomy

Three-port, transconjunctival, sutureless PPV with 27-gauge instrumentation was performed as previously described [2]. The Constellation Vision System (Alcon Laboratories, Inc., USA) was used for all surgeries. In eyes with axial length beyond 30 mm, an extrusion cannula was used to induce a posterior vitreous detachment or the dominant port was removed to allow direct entry of instruments through the sclera to extend the reach of shorter small gauge instruments. Slower fluid egress during indented internal inspection of the peripheral retina was compensated by decreasing the infusion pressure to 5mmHg. In phacovitrectomy cases, phacoemulsification with intraocular lens implantation was completed, using a standard 2.2 mm clear corneal incision approach, before the vitrectomy. In cases where the corneal astigmatism was between 1.0-2.0D, two opposite, clear corneal incisions were placed at the steep axis of the corneal astigmatism or a toric intraocular lens was used if the corneal astigmatism was beyond 2.0D (axis of IOL was confirmed prior to removal of trocars if no tamponade was used and before air infusion if tamponade was used) [9, 10].

No tamponade was used at the end of the surgeries in eyes with no retinal breaks (eyes were filled with balance salt solution). All macular hole surgeries from June 2017 were closed with sulphur hexafluoride gas and an internal limiting membrane flap. A 27-gauge oil cannula (VFI Cannula; MedOne Surgical, Inc.; USA) was used to inject 2000cs oil (Siluron® 2000; Fluoron GmbH; Germany) for all oil tamponade cases. Bimanual active aspiration with oil injection was used when direct heavy liquid – oil exchange was indicated. Oil removal was achieved using an individually packed 25-gauge removable-valve port as third port, with an over-the-port oil removal system, the high-speed extraction sleeve, to optimise speed of removal (Viscous Fluid Control Pak; Alcon Laboratories, Inc., USA).

### Best corrected visual acuity and intraocular pressure

Best corrected distance visual acuity (BCVA) was performed using an electronic test chart (Thompson Software Solution, UK) as previously described [11]. Snellen visual acuity was converted into logarithm of minimum angle of resolution (logMAR). Intraocular pressure (IOP) was measured with the iCare tonometer (iCare, Finland) at pre-operative and post-operative visits.

### Other outcome measures

Operative and post-operative complications reported at any follow-up time point including retinal detachments (in the case of elective surgeries), re-detachments (in the case of retinal detachment surgeries), hypotony, endophthalmitis, limited supra-choroidal haemorrhage and intra-operative iatrogenic detachments were extracted from the electronic patient records. The occurrence and details of any complication was prompted at the time of data entry by the electronic patient record system to maximise data completeness. Anatomic success rates, average surgical time and additional vitrectomies (during the follow-up period) were also recorded.

### Statistical analysis

SPSS® Statistics version 28 (IBM, USA) and Excel 2016 (Microsoft, USA) were used for the statistical analyses. Descriptive statistics was used for continuous and categorical outcomes. Paired-sample t-tests were used to analyse BCVA and IOP for change following 27-gauge vitrectomy. A 5% level of significance was applied.

## Results

### Patient demographics

Table 1 presents the baseline demographic and ocular characteristics of the study population. A total of 958 eyes (consisting of 53.5% right eyes) of 848 patients (consisting of 51.65% females) were included in this study. 4 patients had prior PPV surgeries. Four hundred and four (42.2%) of the 958 primary surgeries were combined with phacoemulsification.

**Table 1:** Baseline and ocular characteristics of the study patients

Variables	Mean (SD) or N(%)
No. of patients	848
Age, years (Mean, SD)	68 (11)
Sex	
Male	410 (48.4%)
No. of eyes treated	958
Study eye	
Right	513 (53.6%)
Lens status	
Phakic	634 (66.2%)
Pseudophakic	317 (33.1%)
Aphakic	7 (0.7%)
Indications for primary surgery	
Macular pucker/Epiretinal membrane	269 (28.1%)
Vitreous haemorrhage and opacities	227 (23.7%)
Retinal Detachment	
Diabetic tractional retinal detachment	5 (0.5%)
Primary RRD	142 (14.8%)
PVR at presentation	21
No PVR at presentation	121
PVR-related recurrent RRD*	1 (0.1%)
Macular hole	145 (15.1%)
Dislocated IOL	21 (2.2%)
Retained lens material	18 (1.9%)
Sub-macular haemorrhage	10 (1.0%)
Vitreomacular traction	113 (11.8%)
Endophthalmitis	7 (0.7%)
Tamponade	
Gas (including air)	272 (28.4%)
Silicone oil	21 (2.2%)
No tamponade	665 (69.4%)
Primary vitrectomy combined with cataract extraction	
Yes	404 (42.2%)

Data displayed are Mean and SD for interval data and % for categorical data PPV, Pars plana vitrectomy; AMD, Age-related macular degeneration; RRD, Rhegmatogenous retinal detachment; PVR, Proliferative Vitreoretinopathy, *primary surgery done elsewhere

### Follow-up duration

The mean follow-up duration of patients following pars plana vitrectomy was 11, standard deviation (SD) 10 months (range: 3–58 months). 573 patients (59.9%) had a minimum of 6 months follow up and 280 patients (29.2%) had a minimum of 12 months follow up.

### Treatment effectiveness

#### Anatomic success rate

Of the 148 retinal detachment cases, 138 (93%) had primary success i.e., required a single operation to treat the detachment. Primary macular hole closure was achieved in 143 of 145 (99%) cases.

#### Best corrected visual acuity

**Fig. 1:**
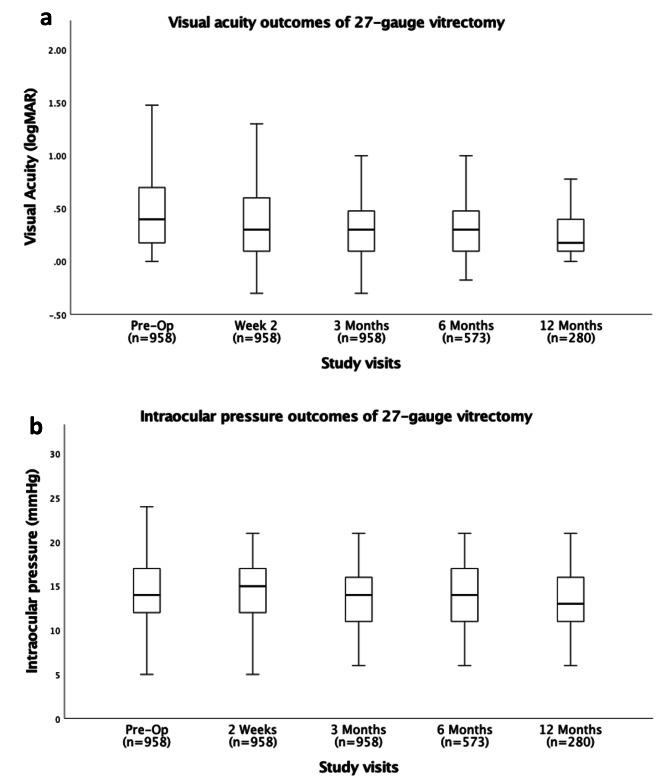
Visual acuity (**a**) and intraocular pressure (**b**) outcomes of 27-gauge vitrectomy

Table 2; Fig. 1a describe the visual acuity of patients pre and post vitrectomy. Overall, there was a significant improvement in BCVA at the final post-operative visit from the pre-op visit [mean logMAR BCVA of 0.59 (SD 0.63) (20/78) and 0.39 (SD 0.52) (20/49) at pre-operative and final post-operative visits, respectively; p < 0.01).

**Table 2:** Visual acuity outcomes of 27-gauge vitrectomy by diagnosis and by tamponade

Diagnosis	n	Pre-op	Day 1	2 weeks	3 months	6 months	12 months
Overall	958	0.59(0.63)	1.14(0.83)**	0.51(0.60)*	0.43(0.54)*	0.44(0.56)*	0.36(0.49)**
*Primary indication*							
RD	148	0.89(0.80)	1.43(0.86)**	0.76(0.71)	0.59(0.61)**	0.63(0.67)	0.52(0.68)**
ERM	269	0.40(0.35)	1.00(0.72)**	0.43(0.40)	0.36(0.37)*	0.37(0.40)	0.27(0.34)*
MH	145	0.56(0.32)	1.51(0.79)**	0.66(0.66)*	0.56(0.61)	0.60(0.75)	0.31(0.32)**
VO	170	0.25(0.40)	0.76(0.79)**	0.17(0.34)*	0.15(0.28)**	0.14(0.26)*	0.17(0.32)*
VMT	113	0.36(0.27)	0.86(0.67)**	0.35(0.35)	0.30(0.32)*	0.33(0.38)	0.35(0.41)
*Tamponade*							
Tamponade		0.73(0.63)	1.47(0.82)**	0.71(0.69)	0.57(0.61)**	0.62(0.70)	0.43(0.56)**
No tamponade		0.52(0.62)	1.00(0.79)**	0.43(0.53)*	0.37(0.49)**	0.37(0.48) **	0.34(0.47)**

Data displayed are Mean (SD) for interval data; RD, Retinal detachment; ERM, Epiretinal membrane; MH, Macular hole; VO, Vitreous opacities; VMT, Vitreomacular traction; *p<0.05; **p<0.01; Test for statistically significant change over time for each post-operative visit is compared with the pre-operative visit

When all the non-RD cases (n = 810) were stratified by whether the vitrectomy was combined with phacoemulsification or not, there was a significant improvement in BCVA at the final visit for both the cases combined with (n = 404) and without (n = 406) phacoemulsification [Mean BCVA of 0.44 (SD 0.44) and 0.33 (SD 0.47) at the baseline and 3-month visits, respectively, for the non-RD cases combined with phacoemulsification, *p* < 0.001; mean BCVA of 0.62 (SD 0.67) and 0.47 (SD 0.56) at the baseline and 3-month visits, respectively, for the non-RD cases without phacoemulsification, *p* < 0.001].

#### Intraocular pressure

Table 3; Fig. 1b describe the IOP of patients pre and post 27-gauge vitrectomy. Overall, the mean IOP was within normal range at the pre-operative and the final post-operative visits (mean IOP of 14.8mmHg and 14.3mmHg at the pre-operative and final post-operative visits, respectively). At the day 1 post-surgery visit, 190 (19.8%) of the eyes that underwent primary vitrectomy had an IOP that was more than 5mmHg different from their pre-operative IOP. 122 (64.2%) of these 190 eyes that had no tamponade, representing 18.3% of the total 665 primary vitrectomy eyes that had no tamponade. The remaining 68 (35.8%) of the 190 eyes were either gas or silicone oil filled at the end of surgery, which also represents a 23.2% of the 293 primary vitrectomy eyes that had tamponades. Although these 190 eyes demonstrated a greater than 5mmHg variance from the pre-operative IOP, most of the eyes had day 1 post-operative IOPs that were between 5mmHg and 21 mmHg. In fact, when all the primary vitrectomies are considered, transient hypotony (≤ 5mmHg) occurred in 39 (4.1%) eyes and 57 (5.9%) eyes had transient ocular hypertension (> 21mmHg) (Table 4).

**Table 3:** Intraocular pressure outcomes of 27-gauge vitrectomy by diagnosis and by tamponade

Diagnosis	n	Pre-op	Day 1	2 weeks	3 months	6 months	12 months
Overall	958	14.79(4.70)	15.13(8.02)	16.97(7.67)**	14.73(5.55)	14.47(4.78)	14.48(5.20)
*Primary indication*							
RD	148	13.68(4.16)	15.82(8.06)**	19.35(10.05)**	15.49(6.52)**	15.05(3.58)*	14.42(3.49)
ERM	269	14.96(4.17)	13.90(7.32)*	17.06(6.71)**	14.79(4.97)	14.60(4.47)	14.84(4.72)
MH	145	15.28(4.02)	16.05(7.91)	16.18(7.32)	14.19(5.37)*	13.36(4.76)*	12.82(3.39)*
VO	170	14.60(3.92)	13.28(7.03)*	16.66(6.42)**	14.63(4.34)	14.78(4.69)	14.36(5.51)
VMT	113	14.76(4.19)	19.06(8.31)**	15.56(7.03)	13.83(5.53)	14.34(6.20)	14.49(7.89)
*Tamponade*							
Tamponade	293	14.47(4.15)	15.93(7.96)*	17.78(8.91)**	14.85(5.99)	14.26(4.23)	13.74(3.49)*
No tamponade	665	14.93(4.91)	14.77(8.01)	16.62(7.03)**	14.68(5.34)	14.55(4.97)	14.71(5.60)

Data displayed are Mean(SD) for interval data; RD, Retinal detachment; ERM, Epiretinal membrane; MH, Macular hole; VO, Vitreous opacities; VMT, Vitreomacular traction; *p<0.05; **p<0.01; Test for statistically significant change over time for each post-operative visit is compared with the pre-operative visit

**Table 4:** Additional vitrectomies within the study period

Surgery	n	Indication for primary surgery (N)	Combined with cataract	Combined with ROSO
Vitrectomy for				
Repair of post-operative retinal detachment secondary to iatrogenic retinal breaks	2	Diabetic retinal detachment (2)		
Repair of new-onset retinal detachment in previously attached retina	2	Vitreous haemorrhage (1) Macular hole (1)		
Retinal re-detachment surgery	17	RRD with PVR (10)	2	5
Silicone oil removal	14	RRD with PVR (14)	2	
Hyphema removal	4	Vitreous haemorrhage (1)		
Vitreous haemorrhage	3	Vitreous haemorrhage (2) Vitreomacular traction (1)	1	
Epiretinal membrane	7	RRD with no PVR (3) RRD with PVR (1) Epiretinal membrane (2) PVR-related recurrent RRD (1)	3	1
Macular hole	4	Vitreomacular traction (2) Avulsed ILM flap during first macular hole surgery (2)	1	
Vitreous opacities	1	Vitreous opacities (1)		
IOL removal from vitreous cavity	2	Sub-macular haemorrhage (1) RRD with no PVR (1)		
Subretinal haemorrhage	5	Subretinal haemorrhage (2)		
Suprachoroidal haemorrhage	1	Dislocated IOL (1)		
Total surgeries	62			

n = number of procedures; N = number of eyes; RRD, Rhegmatogenous retinal detachment; PVR, Proliferative vitreoretinopathy; ILM, Internal limiting membrane; IOL, intraocular lens

#### Additional PPV in the follow up period

Table 5 shows the reason for requiring additional 27-gauge 3 port pars plana procedures during the study period. A further 62 procedures were performed following primary vitrectomy on 42 eyes of 41 patients. 32 eyes had only one additional surgery of which 11 (34.4%) were for silicone oil removal only. Eight eyes had a total of 20 additional surgeries (redo retinal detachment surgeries; final surgeries were for removal of silicone oil). Two eyes had 5 additional surgeries including final removal of silicone oil surgery. Both were single eyed patients with extensive peripheral haemorrhagic macular degenerations involving the entire macula and a pre-surgery vision of hand motion.

**Table 5:** Complications of 27-gauge vitrectomy

Complications	n	Primary indications
**Intraoperative**		
Retinal breaks	2	Diabetic traction retinal detachment (2)
Suprachoroidal haemorrhage	1	
**Post-operative**		
Retinal detachment in previously attached retina	2	Vitreous haemorrhage (1) Macular hole (1)
Transient ocular hypertension (> 21mmHg at day 1)	57	
*Ocular hypertension by indication for primary vitrectomy*:		
Retinal detachment	10	
Epiretinal membrane	9	
Macular hole	11	
Vitreous haemorrhage	5	
Vitreous opacities	8	
Vitreomacular traction	10	
Other indications	4	
*Ocular hypertension by tamponade*:		
No tamponade	36	
Gas	18	
Silicone oil	3	
Transient ocular hypotony (≤ 5mmHg at day 1)	39	
*Ocular hypotony by indication for primary vitrectomy*:		
Retinal detachment	5	
Epiretinal membrane	9	
Macular hole	7	
Vitreous haemorrhage / opacities	7	
Vitreomacular traction	3	
Other indications	8	
*Ocular hypotony by tamponade*:		
No tamponade	27	
Gas	10	
Silicone oil	2	
Epiretinal membrane	7	

Data displayed are Mean(SD) for interval data; RD, Retinal detachment; ERM, Epiretinal membrane; MH, Macular hole; VO, Vitreous opacities; VMT, Vitreomacular traction; *, *p* < 0.05; **, *p* < 0.01; Test for statistically significant change over time for each post-operative visit is compared with the pre-operative visit

In terms of silicone oil removal, a total of 17 eyes of 16 patients underwent 20 additional procedures to remove silicone oil. Twelve of these eyes had a standalone silicone oil removal following a primary vitrectomy for rhegmatogenous retinal detachment (RRD) with proliferative vitreoretinopathy (PVR). Another eye, previously treated for a primary indication of RRD with PVR and had developed epiretinal membrane, underwent a combined procedure of silicone oil removal and membrane peeling. Four other eyes underwent removal of silicone oil in combination with surgical repair of retinal redetachment under silicone oil. Two out of these 4 eyes had additional procedures to remove the silicone oil. Another eye out of the 4 eyes had a further silicone oil removal combined with surgical repair of a recurrent retinal detachment under silicone oil.

#### Complications

Table 4 describes the complications resulting from pars plana vitrectomy. There were two diabetic delamination cases that developed iatrogenic intraoperative retinal detachment during air to fluid exchanges, prior to setting an infusion delay and decreasing flow. One other eye (high myopia of -16.0D) developed a vitreous bleed from a limited intraoperative suprachoroidal haemorrhage (see discussion for details). Seven eyes developed epiretinal membrane during the follow-up period, which were treated with a second surgery.

#### Surgical time

The average surgical times were 55 min for retinal detachment surgeries and 38 min for all other indications.

## Discussion

This retrospective study is the first that has investigated the exclusive use of 27-gauge vitrectomy for all indications on a large cohort of 1020 vitrectomies performed on 958 eyes to determine any operative and post-operative complications associated with the procedure. The findings of this study indicate that 27-gauge instrumentation can be safely and effectively used for vitrectomy on all the included vitreoretinal indications.

Surgeries performed with smaller incisions are associated with reduced scarring, increased accuracy, and lower risks of encountering intra- and post-operative complications [1, 2, 5]. The advantages and disadvantages of transitioning from 23-gauge to 25-gauge instruments are amplified with a progression from 25-gauge to 27-gauge instruments [12, 13]. The small distance from the vitrector tip to the port facilitates aspiration of membranes (fibrovascular membranes for diabetic delaminations, epiretinal and PVR membranes after starting posteriorly with forceps) and retina during retinectomies. This functionality is further enhanced with a bevelled vitrectomy tip and the smaller sphere of influence associated with 27-gauge which reduces distant vitreoretinal traction effects and a reduced risk of inadvertent retinal trauma [12, 14]. The additional advantages of using a single-gauge platform lie in the lack of confusion as to what gauge instruments to set-up for each case and the improved procurement or supply-chain processes.

Initial concerns with 27-gauge vitrectomy included increased operation time [7]. It is worth mentioning that the efficiency of vitrectomy is dictated by the laws of physics such that if all other parameters are kept constant, it would be impossible to remove vitreous quicker through a smaller bore instrument [15]. Typically, no additional manoeuvres are required for some indications with good prognosis (e.g., vitrectomy for vitreomacular traction and removal of vitreous haemorrhage or opacities). As a result, the advantages of smaller gauge instruments for such cases (including more stable post-surgery IOP and less eye wall trauma) may outweigh an acceptable increase in surgical duration. For indications where additional manoeuvres are required, vitrectomy itself is only one part of the surgery in order to achieve the surgical goal. Hence, any time lost in the vitrectomy portion of such surgeries will be diluted by the time saving or time neutral portion of the additional manoeuvres [16, 17]. This is supported by the findings of Li and colleagues who observed no significant difference in surgical time between 27-gauge vitrectomy and 25-gauge vitrectomy with silicone oil for primary RRD [18]. Therefore, the discussion around efficiency of 27-gauge instruments, when compared with 25-gauge instruments, must be one of either “acceptable decrease” in efficiency or a trade-off between the above-mentioned improvements for a given decrease in efficiency. It is also important to note that improvements, as described here, do not equate to clinical significance.

The evidence reported in this study is consistent with previous studies and suggests that it is possible to use only 27-gauge instrumentation for all vitrectomy indications. The overall visual acuity outcomes are also comparable with previous reports [2, 19]. In a study by Khan and colleagues, which investigated the long-term and safety profile of 27-gauge vitrectomy, the overall mean visual acuity improved from 20/105 to 20/50 [2]. Similarly, Yoneda and associates showed an improvement in best corrected visual acuity, in a retrospective study that assessed the safety and efficacy of 27-gauge vitrectomy for consecutive series of 163 eyes, from 20/58 at the pre-operative visit to 20/32 at the final visit [20].

The most striking data to note was the stability of intraocular pressure immediately after surgery. Our finding of stable IOP is similar to the results from previous 27-gauge vitrectomy studies [2, 20, 21]. While changes in IOP from pre-surgery to Day 1 post-surgery have been shown to be more variable with larger gauges, a recent meta-analysis conducted by Ma et al., has shown that the Day 1 post-operative IOP in the 27-gauge group was comparable with that of the 25-gauge group [7, 12, 22]. Although the Day 1 post-surgery was stable with both 27-gauge and 25-gauge instruments, a careful drill through of the data actually shows that the rate of hypotony (out of 6 studies reporting hypotony) was 3.3% and 7.3% in the 27-gauge and 25-gauge groups, respectively [12]. A study by Naruse et al., which compared 27-gauge and 25-gauge vitrectomy for idiopathic epiretinal membrane, showed that IOP was more stable in the 27-gauge group than the 25-gauge group, with hypotony rates of 2% and 6% in the 27-gauge and 25-gauge groups, respectively [23]. Post-operative hypotony in their study was found to be caused by post-operative subclinical leakage [23]. The smaller incision size of 27-gauge improves the self-sealing nature of the sclerostomies and is also likely responsible for the non-occurrence of endophthalmitis in this case series [24].

Overall, we found that 27-gauge surgery was not associated with a poorer retinal detachment surgery outcome. Our anatomic success rate of 93% is similar to previously reported results [2]. In addition, we observed a low rate of complications for other vitrectomy indications, in keeping with prior reports [25]. The macular hole closure rate observed in the present study is consistent with previous reports [26]. Both failed macular hole surgeries occurred during the first 6 months of conversion to ILM flap surgeries for all macular holes where the ILM flap was recorded as “avulsed” at time of surgery. Our subsequent high closure rate is likely due to our relatively small sample size and the closure of 2 large macular holes (larger than 1000 microns) with primary full thickness retinal patch grafts.

In this series, a single suprachoroidal haemorrhage occurred in a highly myopic eye within the first 200 surgeries. The Constellation Vision system used in this study is designed to match the disparity between the pressure in the eye to a surgeon’s pre-set infusion pressure by actively pressurising the infusion to compensate for the pressure gradient during aspiration and reducing the occurrence of hypotony [27]. In air filled eyes, when returning to fluid and during air aspiration, this discrepancy is largest, and the infusion jet is of a higher flow rate because the bore of the infusion is smaller. This extremely high flow rate can cause a rapid jet of fluid to the retinal surface, which can in turn cause retinal break formation as occurred in two patients early on in this series. The Constellation system can be configured to prevent this rapid increase in infusion flow with IOP compensation activated, by means of a compensation delay time and this is crucial particularly in 27-gauge surgery.

Fluid does not egress the eye as quickly with a 27-gauge infusion compared with larger gauge infusions [28]. Therefore, in an attempt to keep patients comfortable during careful, indented internal search for iatrogenic breaks, the infusion pressure was decreased to 5mmHg (in addition to supplementary subconjunctival anaesthesia 5 min prior to indentation) in all cases up to the time that the one eye in our series developed a suprachoroidal haemorrhage. We believe the haemorrhage may have been prevented by using a higher infusion pressure. Following this, the infusion pressure was lowered to 10mmHg only (instead of 5mmHg) prior to indentation with no further haemorrhages.

The lack of rigidity of instruments is a well-known limitation to adoption of 27-gauge surgeries [27]. The 27-gauge plus system used, has a stiffening sleeve on the proximal vitrectomy probe shaft and in addition the surgeon utilised ‘finger buttressing’ at the junction between the instrument shaft and the instrument handle during surgery to increase instrument rigidity. Newer designs of instruments with stiffer materials and longer self-retracting sleeves between the shaft and the handle of instruments may address this issue further in the future.

A limitation of this study is the retrospective, non-comparative nature of the study design. More prospective trials comparing 27-gauge instrumentation with other gauges are warranted to enhance our understanding of the safety and efficacy profile of 27-gauge instrumentation. Further, the duration of the vitrectomy portion of surgeries were not specifically recorded. Hence, the present study cannot conclude on whether the 27-gauge vitrectomy time for the subtypes of surgical indications was shorter or longer and whether the vitrectomy time was comparable to other gauges [16]. This aside, a higher proportion of cases were combined with cataract surgery, a common practice pattern outside of the United States [29]. This may potentially influence the visual acuity outcomes and may not entirely reflect the visual function treatment outcomes of the vitrectomy procedure. It is also worth mentioning that the case mix included in this study may not be representative of other practices. For example, RRD cases as well as complex cases including PVR, complex diabetic tractional retinal detachments and trauma cases, were relatively uncommon in this study compared with other studies [30]. Another limitation of our study is the absence of data on how many ports were sutured. However, in the same way a decrease in flow through a smaller tubing is dictated by the law of physics, the rate of self-sealing wound is also intuitively better with smaller gauges. Our clinically relevant IOP data alludes to this.

## Conclusion

The findings of this study indicate that it is possible to use 27-gauge vitrectomy instruments for a wide range of indications, with exclusive use in certain settings as presented in this series. The outcomes were similar to other gauges, including in rhegmatogenous retinal detachment, with a low rate of complications.

## Data Availability

The datasets used and/or analysed during the current study are available from the corresponding author on reasonable request.
